# An Extremely Rare Reason for Failure of Left Sided Pacemaker Implantation

**DOI:** 10.1155/2014/453071

**Published:** 2014-11-19

**Authors:** Erdem Özel, Ali Öztürk, Emin Evren Özcan, Berhan Genç

**Affiliations:** ^1^Cardiology Department, Şifa University, 35540 İzmir, Turkey; ^2^Radiology Department, Şifa University, 35540 İzmir, Turkey

## Abstract

We reported a case of isolated anomaly of the left brachiocephalic vein which is diagnosed during a permanent pacemaker implantation. It is a very rare anomaly and makes the left sided pacemaker implantation impossible.

## 1. Introduction

Anomalies of the great thoracic veins are uncommon [1]. Vast majority of anomalies of these veins are diagnosed on routine radiologic examinations incidentally [2]. Persistent left superior vena cava (LPSVC) is the most common systemic venous anomaly of thorax with the incidence of 0,5% [3]. However isolated anomaly of the left brachiocephalic vein (LBCV) is extremely rare [4]. Anomalies of the LBCV are clinically silent unless accompanying cardiac defects, but the anomalies of the LBCV might pose a challenge if invasive procedures are scheduled to be driven through this route. Herein we report a failed left sided pacemaker implantation attempt due to isolated anomaly of the LBCV.

## 2. Case Presentation

A 77-year-old woman with the diagnosis of sick sinus syndrome was referred to our institution for permanent pacemaker implantation. Left pectoral site was prepared for implantation according to routine practice. Left subclavian puncture was performed successfully. But we could not advance the guidewire into the LBCV. Instead of getting into LBCV, the guidewire made a sharp turn and propagates downwards after subclavian vein. The repetitive attempts showed the same result. On cine venography, an accessory hemiazygos vein was seen which takes blood from LBCV and drains into azygos vein. Because of the challenging anatomy, we decided to make the implantation from the right side. And at the same session, we implanted DDD-R (Zephyr DR Saint Jude Medical, USA) permanent pacemaker by using the right subclavian vein, without difficulty.

Then we performed a multidetector thorax CT angiography to see real venous anatomy and accompanying defects clearly.

**Figure 1:**
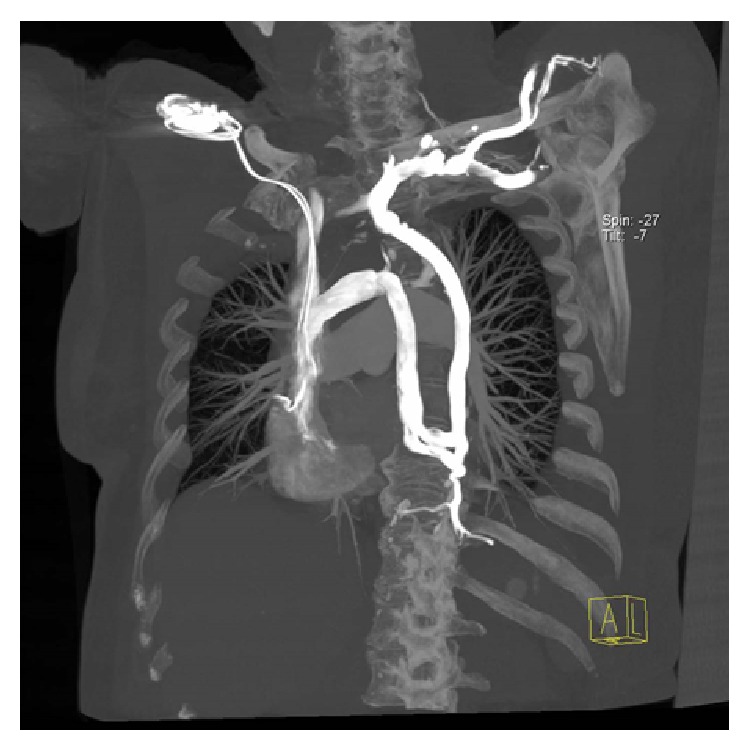
Reformatted CT angiography image shows the anomaly in the venous phase.

**Figure 2:**
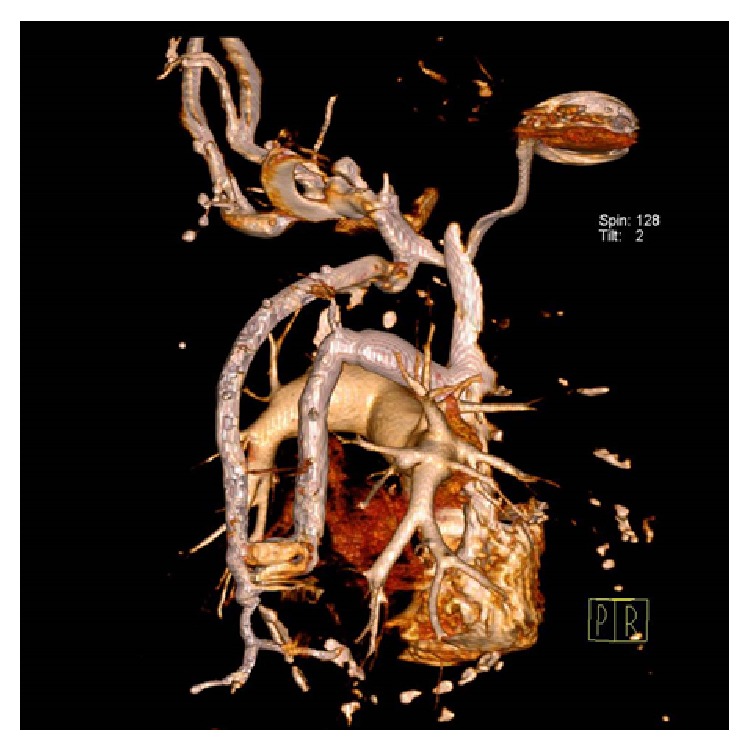
Volume rendering CT image shows the anomaly from the backside.

CT angiography (Figures 1 and 2) revealed that LBCV was draining into the accessory hemiazygos vein and this accessory hemiazygos vein was connecting with the hemiazygos at the level of tenth thoracic vertebrae and both were draining into left superior vena cava via azygos. Based on this identified anomaly the azygos system was dilated. And also CT angiography showed elongation and dilatation of the ascending aorta.

## 3. Discussion

Venous anomalies of the thorax involve pulmonary and systemic veins. They might be encountered on a broad spectrum. Spectrum can range from simple incidental finding on radiographic procedures to complex venous anomaly generally accompanying congenital heart disease [3].

Most common anomaly of the pulmonary veins is partial anomalous of pulmonary venous return. The prevelance of this anomaly is 0,3% in autopsy series [5].

The most common venous anomaly of the thorax is LPSVC. And this is the major congenital venous anomaly, which precludes left sided pacemaker implantation. Despite the existence of this anomaly, implanting cardiac device successfully is reported in the literature [6, 7]. However, total absence of LBCV is an extremely rare condition and left sided cardiac device implantation is impossible in this situation. But as we saw in this case, right sided implantation can be done safely.

Although we implanted the permanent pacemaker successfully from the right side at the same session, repeated punctures can raise the risk of pneumothorax. So, after failed attempts from the left side, a chest X-ray can be performed and procedure can be deferred.

To avoid multiple subclavian vein punctures, peripheral venography can be done before the procedure. This will help to differentiate common venous anomalies and decrease the risk of access site complications. We do not perform routine peripheral venography before pacemaker implantations in our institute, but in this case it would be extremely helpful in detecting the venous anomaly and managing the procedure.

In conclusion physicians who deal with thoracal venous procedures must be familiar with venous anomalies of the thorax. And the absence of LBCV is a very rare reason which makes left sided cardiac device implantation impossible.
